# Socioeconomic variations in nicotine dependence in rural southwest China

**DOI:** 10.1186/s12889-015-2492-9

**Published:** 2015-11-23

**Authors:** Le Cai, Wenlong Cui, Dingyun You, Jianhui He, Keying Zhao

**Affiliations:** Cheng Gong New City, School of Public Health, Kunming Medical University, 1168 Yu Hua Street Chun Rong Road, Kunming, 650500 China

**Keywords:** Smoking, Nicotine dependence, Socioeconomic status, Multilevel analysis, China

## Abstract

**Background:**

This study examines how nicotine dependence is distributed across socioeconomic gradients in rural Yunnan province, which has the most ethnic minorities in one province in southwest China.

**Methods:**

A cross-sectional survey was conducted in four rural areas of Yunnan province among 17,158 consenting individuals aged ≥18 years in 2011. Information on demographic characteristics and smoking habits was obtained using a standard questionnaire. The Fagerstrom Test for Nicotine Dependence (FTND) was applied to assess nicotine dependence. Multilevel logistic regression was used to model the variation in prevalence of nicotine dependence.

**Results:**

In the study population, the overall prevalence of current smokers and nicotine dependence was 32.4 % and 31.6 %, respectively. Females were much less likely to have nicotine addiction than males: odds ratio (OR) of 0.01 (95 % CI: 0.008 – 0.012). Higher annual household income was associated with a greater risk of nicotine dependence (OR 1.09, 95 % CI: 1.01 – 1.17). Adults who grew tobacco were more likely to have nicotine addiction (OR 1.22, 95 % CI 1.07 – 1.41). Individual educational level was inversely associated with the probability of nicotine dependence (OR 0.63, 95 % CI 0.55 – 0.72), lower community educational level was also associated with an increased risk of nicotine dependence (OR 0.94, 95 % CI 0.92 – 0.98).

**Conclusions:**

Nicotine dependence showed significant variations across different indicators of both contextual and individual socioeconomic status in rural southwest China. Future interventions on tobacco cessation should give increased attention to men, tobacco farmers, less educated or poorer persons, and lower educational level communities.

## Background

Smoking is well known as the single most preventable cause of death and disability in the world. The World Health Organization (WHO) estimates that the number of smokers is expected to increase from 1.3 billion to 1.7 billion worldwide by the year 2025 [1], with particularly serious health impacts in developing countries [2]. China is both the world’s largest producer and consumer of tobacco products, with an estimated 301 million current smokers in 2010 [3]. Despite the high prevalence of smoking, there is a low rate of attempting to quit smoking among current smokers. In rural China, particularly among men [4], nicotine dependence has become a significant public health problem.

A better understanding of the prevalence and determinants of nicotine dependence is vital for facilitating the development and implementation of effective tobacco control interventions. Studies have identified that individual level socioeconomic status (SES) factors like age, sex, income, and educational level are the main determinants of nicotine dependence [5–7]. Being male, low level of education and income are associated with higher nicotine dependence. However, little is known about the association between contextual SES and nicotine dependence.

In China, a few studies also have found an inverse association between education and nicotine dependence [8–10]. However, studies examining prevalence and determinants of nicotine dependence in China have focused on urban areas while overlooking rural communities. Furthermore, the association between contextual SES and nicotine dependence is still poorly understood in China. Therefore, factors at both the individual and contextual level that can influence the susceptibility of individuals to nicotine dependence need further attention.

China is a multi-ethnic country and has 56 ethnicities. The Han ethnic is the majority (>92 %) and the remaining 55 ethnicities are the national minorities. Yunnan province is located in southwestern China and had a recorded population of 45.9 million people in 2010, among which rural communities accounted for 70.5 % of total population [11]. Yunnan contains the most ethnic minorities in China, with 25 different ethnic minorities living in the province. Ethnic minorities account for 38.07 % of the region’s total population. Yunnan is known as a tobacco kingdom in China, with over 70 % of the local tax income coming from the tobacco industry. Although smoking has been highly prevalent in rural Yunnan, particularly among men [4], nicotine dependence rates and its SES determinants are not well documented in this region.

In Yunnan, proportion of ethnic minorities and area of tobacco cultivation are useful variables to describe the characteristic of a community. Other variables like population size, adult literacy rate, and average income are also frequently used as generic indicators of contextual socioeconomic characteristics in Chinese epidemiological studies [12]. They are census variables. Therefore, the aim of this study was to apply multilevel regression analysis to simultaneously investigate how nicotine dependence varied across a range of indicators of both individual and contextual SES among the rural adult population of southwest China in 2011.

## Methods

### Study design, subjects and sampling techniques

This study was a community-based, cross-sectional survey conducted in four rural areas of Yunnan province, China. A multi-stage stratified random sampling method was used to select individuals aged ≥18 years from a total of 44 townships, details of the sampling methods have been described elsewhere [13].

### Data collection and measurement

Before being interviewed each participant was given a full explanation of the research project and its purpose. If the respondent agreed to participate in the study, he or she was individually interviewed face-to-face by trained interviewers using a pre-tested and structured questionnaire. The questionnaire asked about individual demographic characteristics, household income, educational level, self-reported smoking habits, and behavioral practices. Self-reported current smokers were assessed for nicotine dependence using six questions from the Fagerstrom Test for Nicotine Dependence (FTND) [14]. The FTND has a set of 6 items with an overall score ranging from 0–10.

Socioeconomic characteristics of 44 townships for the year 2011 were obtained from the local statistics office.

### Consent statement

Each participant signed an informed consent form before being interviewed.

### Ethical approval

This study was approved by the Ethics Committee of Kunming Medical University, before carrying out the research.

### Definitions

Smokers were defined as persons who had smoked at least 100 cigarettes in their lifetime, and those who smoked on a daily basis at the time of the survey were classified as current smokers. Very high dependence was defined as an FTND score ≥8; high dependence, a score ranging from 6–7; medium dependence, a score equal to 5; low dependence, a score ranging from 3–4; and very low dependence, a score ranging from 0–2; no-dependence, a score <5; For this study, dependence was defined as a score ≥5.

### Statistical analysis

Multilevel logistic regression was used to analyze the association between both contextual and individual SES variables and a binary measure of nicotine dependence. Individual characteristics were: age, sex, ethnicity, yearly household income, education, and tobacco cultivating status. The township characteristics or contextual variables were the percent middle (grade 7–9) or higher education completed percentage of ethnic minorities, average yearly income, population size, area of township, and area of tobacco cultivation. Data analyses were done with R software version 2.9.2 [15].

## Results

In total, 18,000 individuals from the forty-four townships were invited to participate in the survey from among the eligible individuals. Of these 17,158 consented to participate, for an overall response rate of 95.3 %.

Table 1 shows the demographic characteristics of the study participants. Participants in the study included 8156 males and 9002 females. Among the study participants, 30.9 % were ethnic minorities, and 19.4 % were engaged in tobacco cultivation. Males had higher educational level than females (*P* < 0.05). In the 12 months before the survey, only 17.9 % of current smokers tried at least one attempt to quit smoking. 81.2 % of current smokers initiated smoking during adolescence. The mean FTND score was 3.4 (3.4 for males and 3.3 for females).

**Table 1:** Individual characteristics of the study population

Characteristics	Male (*n* = 8156)	Female (*n* = 9002)	All (*n* = 17158)
Age (%)			
< 35 years	1966 (24.1)	2098 (23.3)	4064 (23.7)
35–44 years	1909 (23.4)	1911 (21.2)	3820 (22.3)
45–59 years	2186 (26.8)	2576 (28.6)	4762 (27.8)
≥ 60 years	2095 (25.7)	2417 (26.8)	4512 (26.3)
Ethnicity (%)			
Han	5632 (69.1)	5983 (66.5)	11615 (67.7)
Minority	2524 (30.9)	3019 (33.5)	5543 (32.3)
Level of education (%)			
Primary (grade 1–6) or lower	6723(82.4)*	7876 (87.5)	14599 (85.1)
Middle (grade 7–9) or higher	1433 (17.6)	1126 (12.5)	2559 (14.9)
Approximate yearly household income (US$)			
Mean	807.6	688.4	746.0
Min-Max	39.7-9539.0	47.7-2861.7	39.7-9539.0
Tobacco cultivating (%)	1707 (20.9)	1620 (18.0)	3327 (19.4)
Age of smoking initiation			
< =11 years	135 (2.5)**	2 (1.8)	137 (2.5)
12–20 years	4371 (80.1)	61 (55.5)	4432 (79.6)
> =21 years	956 (17.5)	42 (38.2)	998 (17.9)
Previous attempts to quit (%)			
At least one attempt in previous 12 months	987 (18.1)	12 (10.9)	999 (17.9)
No attempt	4470 (81.9)	98 (89.1)	4568 (82.1)
Mean FTND score	3.4 ± 2.2	3.3 ± 2.1	3.4 ± 2.2

*P < 0.05, **P < 0.01

Table 2 presents socio-demographic correlates of current smoking and nicotine dependence among study participants. The overall prevalence of nicotine dependence was 31.6 % based on FTND ≥ 5, and a total of 18.5 % of current smokers were highly nicotine addicted. Males had remarkably higher prevalence of current smokers and nicotine dependence than females (*P* < 0.01). The highest rates of current smokers and nicotine dependence were found among individuals aged 35–44 years. The Han ethnicity had higher prevalence of current smokers than ethnic minorities (*P* < 0.05). Prevalence of nicotine dependence decreased as level of education increased (*P* < 0.05), whereas prevalence of current smokers and nicotine dependence increased with increased level of yearly household income (*P* < 0.01). Individuals who cultivated tobacco had a higher prevalence of current smokers and nicotine dependence than those who did not (*P* < 0.01).

**Table 2:** Socio-demographic correlates of current smoking and nicotine dependence in rural southwest China

Variables	Current smokers	Nicotine dependence		
		Low or very low dependence	Medium dependence	High or very high dependence
	n (%)	n (%)	n (%)	n (%)
Sex				
Male	5457 (66.9)**	3735 (68.4)	710 (13.0)	1012 (18.5)**
Female	110 (1.2)	91 (82.7)	11 (10.0)	8 (7.3)
Age				
< 35 years	1231 (30.3)	917 (74.5)	142 (11.5)	172 (14.0)
35–44 years	1404 (36.8)**	906 (64.5)	177 (12.6)	321 (22.9)**
45–59 years	1564 (32.8)	1001 (64.0)	228 (14.6)	335 (21.4)
≥ 60 years	1368 (30.3)	982 (71.8)	184 (13.5)	202 (14.8)
Ethnicity				
Han	3845 (33.1)*	2671 (69.5)	494 (12.8)	680 (17.7)
Minority	1722 (31.1)	1135 (65.9)	237 (13.8)	350 (20.3)
Level of education				
Primary (grade 1–6) or lower	4718 (32.3)	3198 (67.8)*	644 (13.6)	876 (18.6)
Middle (grade 7–9) or higher	849 (33.2)	608 (71.6)	87 (10.2)	154 (18.1)
Level of yearly household income (US$)				
Low (<810)	4214 (31.4)**	2881 (68.4)	565 (13.4)	768 (18.2)*
High (≥810)	1353 (36.2)	925 (68.4)	166 (12.3)	262 (19.4)
Tobacco cultivating				
Yes	1293 (38.9)**	847 (65.5)	151 (11.7)	295 (22.8)**
No	4574 (30.9)	2959 (69.2)	580 (13.6)	735 (17.2)
All	5567 (32.4)	3806 (38.4)	731 (13.1)	1030 (18.5)

*P < 0.05, **P < 0.01

Table 3 displays the township contextual variables among the forty-four townships. There were high overall variations in the percent middle (grade 7–9) or higher education level completed, percentage of ethnic minorities, average yearly income, population size, area of township, and area of tobacco cultivation among the 44 townships.

**Table 3:** Distribution of socioeconomic status for the 44 townships

Variables	Min	P_25_	P_50_	P_75_	Max
Percent middle (grade 7–9) or higher education level (%)	60.5	81.9	85.4	89.5	95.3
Percentage of ethnic minorities (%)	0.05	3.1	11.3	82.2	98.8
Average yearly income (1000 US$)	0.26	0.45	0.65	0.79	1.45
Population size	5665	10709	21687	62846	120243
Area of township (Square kilometers)	12	110	208	307	574
Area of tobacco cultivation (Square kilometers)	0	17	41	114	384

Table 4 shows the results of multilevel analysis of the effect of both townships and individual characteristics on nicotine dependence. Males had much higher probability of being nicotine addicted than females. Higher annual household income was associated with a greater risk of nicotine dependence. Adults who grew tobacco were more likely to be nicotine addicted. Both individual and township educational level was negatively associated with nicotine dependence.

**Table 4:** Odds ratios (OR) and 95 % confidence intervals (CI) for multilevel logistic regression analysis of nicotine dependence in rural southwest China

Predictors	Nicotine dependence (reference: non-nicotine dependence)	
	OR	95 % CI
Individual variables:		
Sex (reference: male)	0.01**	(0.008, 0.012)
Education level (reference: middle (grade 7–9) or higher)	0.63**	(0.55, 0.72)
Level of yearly household income (reference: low)	1.09*	(1.01, 1.17)
Tobacco cultivation (reference: no)	1.22*	(1.07, 1.41)
Contextual variables:		
Percent middle (grade 7–9) or higher (reference: primary (grade 1–6) or lower)	0.94*	(0.92, 0.98)

*P < 0.05, **P < 0.01

## Discussion and conclusions

The findings showed that smoking and nicotine dependence were highly prevalent among men in rural southwest China. The phenomenon that men smoked much more and were more likely to have nicotine addiction than women was also found in other Chinese studies [3, 4, 8, 10]. Furthermore, the prevalence of smoking and nicotine dependence among men in the region under study was greater than the prevalence rate observed in urban Chinese populations [8] as well as studies from Singapore [16], India [7], and Sri Lanka [17]. Observed prevalence of smoking and nicotine dependence were also comparable to findings reported in previous European study [6], but had a much lower prevalence of current smokers who have previously attempted to quit smoking observed by other Chinese and Western studies [18, 19]. The findings indicate that smoking and nicotine dependence in males is a serious public health concern in rural southwest China.

Our study found that individuals who cultivate tobacco have higher rate of current smokers and nicotine dependence than individuals who do not. Tobacco cultivating status has been demonstrated as an important determinant of smoking and nicotine dependence in a previous Chinese study [4]. The finding suggests tobacco farmers could be the target of future tobacco control interventions in rural China.

In the study population, individual educational level was inversely associated with the probability of nicotine dependence. The negative association between individual educational level and nicotine dependence has also been found in other Chinese and Western studies [9, 20–22]. Furthermore, our study indicated that contextual level of education was also associated with a protective effect on nicotine dependence. Living in communities with higher overall educational levels was associated with a decreased risk of nicotine dependence. This is possibly due to the fact that influences of peers and neighbourhood on smoking and social activity are very strong in Chinese communities. People with higher levels of education tend to have a healthier lifestyle. The strong association between both contextual and individual educational level and risk of nicotine addiction in our study suggests that implementing robust smoking cessation programs to reduce tobacco use in rural China should focus on less educated people in parallel with those living in communities with lower levels of education.

In the present study, higher individual annual income showed a significant positive association with nicotine dependence. This result is consistent with previous Chinese studies [4], but differed from some western studies where individuals with lower incomes are more likely to be nicotine addicted [23, 24]. Perhaps persons with higher incomes are more able to affordably purchase tobacco products and therefore consume more.

The current study had the following limitations. First, prevalence of smoking was based on self-reporting, and may therefore be subject to recall bias. Second, the present findings were based on a study of random sampling of four counties, which may limit the ability to generalize the results to the whole province.

In conclusion, this study shows a high prevalence of smoking and nicotine dependence and low level of attempting to quit smoking among current smokers in rural southwest China. Additionally, nicotine dependence varies by both contextual and individual SES variables. These findings highlight the need for tobacco cessation interventions to target men, tobacco farmers, less educated or poorer persons, and lower educational level communities.

## Availability of data and materials

Data will not be shared, because this research was funded by the Bill & Melinda Gates Foundation and Major Achievement Cultivation Project Fund of Kunming Medical University. It is not allowed to share dataset with others without permission.
